# Supplementary material for: Curating genomic disease-gene relationships with Gene2Phenotype (G2P)
Source: Genome Med. 2024 Nov 6;16:127. doi: 10.1186/s13073-024-01398-1 (PMC11539801; doi:10.1186/s13073-024-01398-1)
Supplement: Supplementary file 2 — Additional file 2. G2P Gene Record version 7.3 Curation SOP. [file 13073_2024_1398_MOESM2_ESM.docx]

## G2P Gene Record version 7.3 Curation SOP

This SOP describes the manual curation procedure followed by the Developmental Disorders G2P (DDG2P) team of clinical and scientific curators. The curation process starts with the critical and in-depth assessment of the relevant peer-reviewed scientific literature, followed by the recording of the genetic and molecular mechanisms underlying the gene-disease association using standardised terminologies. This information is then used, in clinician-led bimonthly curation meetings, to discuss the gene-disease association supporting evidence and assign an agreed level of confidence to the gene-disease assertion.

**Table of contents**

[1. Create a new G2P curation record 2](#_uri8zny2i2mp)

[2. Locus 2](#_snoz910ab6q)

[3. Publications 2](#_icqfv2eyh04s)

[4. Background (or Additional) information 3](#_nijdw2nsb1nb)

[5. Clinical phenotype 3](#_pnffuc6fem5n)

[6. Genotype 4](#_pkibzsey44cm)

[6.1. Allelic requirement 4](#_vr0nka2tl57)

[6.2. Cross cutting modifier 4](#_nhfhme17wtl8)

[6.4. DECIPHER Protein View 5](#_n415a9utjwza)

[6.5. Variant consequence per allele for relevant allelic requirement 5](#_qruph71z0epd)

[7. Mechanism 6](#_n7x2az28z7g4)

[7.1. Synopsis of mechanism 6](#_ghq0512rqjte)

[8. Additional comments 6](#_11xhg9wutgg7)

[9. Disease entity 7](#_5371fon7yrmi)

[9.1. Disease name 7](#_2v33nxdjjcn2)

[9.1.1. Existing disorders 7](#_t51bjbvb9uv6)

[9.1.2. New disorders 7](#_hon9keuv3fav)

[9.2. Panel 7](#_g3jkooli1l7t)

[9.3. Agreed confidence category 8](#_ao4lbt5knhzo)

[Appendix 9](#_dxbwvn16blmv)

[Table 1. HPO allelic requirement terms 9](#_i2u5kx4uq2mr)

[Table 2. HPO inheritance qualifier terms. 9](#_6ekbav4hedoo)

[Table 3. Sequence Ontology (SO) type of variant terms. 10](#_buv0cjcwdp2n)

[Table 4. Sequence Ontology (SO) functional effect variant terms. 11](#_a4st8jytiige)

[Table 5. Description of G2P confidence categories. 11](#_z7noz31ktejb)

### 1. Create a new G2P curation record

Once a gene-disease association has been selected for curation based on the potential relevance for G2P, create a new curation record by adding the following information:

**Version:** Record version of the curation record (version 1). Version will change if the document is revised after it has been discussed at the curation meeting.

**Date created:** Record date the curation is started.

**Date modified:** If there are changes to the curation record after it has been discussed at the curation meeting, please add the date when the changes are made.

### 2. Locus

This is a mandatory and unique field.

**Gene symbol (HGNC):** add the approved HGNC symbol for the gene being curated. NB the gene symbol is used here for ease of use. This is then mapped to the stable HGNC ID.

### 3. Publications

This is a mandatory field.

The curation of a new gene-disease association starts with the identification of one or more relevant peer-reviewed publications. Perform a check of Europe PMC to identify additional relevant publications. Search strategies may include {gene symbol}, {gene symbol}[TI] (for symbol in title), {gene symbol}[TIAB] (for symbol in title or abstract), {disease name}, {disease name} AND {gene symbol}. All publications used in the curation record must be listed in the **List of associated PMID and titles.**


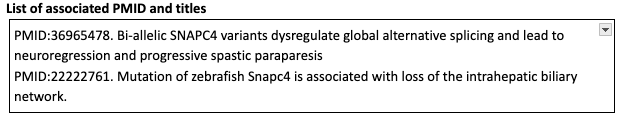


For each publication, annotate the number of families reported and the associated phenotypes in the **Number of families and associated phenotype per PMID** section**.** If relevant, annotate the consanguinity status and specific ethnicity when it is reported.

| **PMID** | **No. of families** | **Notes e.g. phenotypic features, variants reported** |
| --- | --- | --- |
| 22748208 | 1 (2 sibs) | Developmental regression, microcephaly, nonverbal, optic atrophy, cortical atrophy, thin corpus callosum. Skin biopsy NCL-type lysosomal storage material. |
| 30500434 | 2 | Patient 1 epilepsia partialis continua, myoclonic seizures, atypical absences, deterioration in cognitive and motor function. No NCL on skin biopsy. Patient 2 Regression, myoclonic seizures, NCL features on skin biopsy. |
| 32412666 | 1 | Cognitive and motor deterioration, ataxia, epileptic paroxysms, and MRI signs of cerebellar atrophy. |

### 4. Background information

To better understand the biological context of the gene-disease association, copy the UniProt protein function description either from the DECIPHER overview or the UniProt Function and add the text in the **Protein function as described by UniProt** section**.**

Review current disease associations for the curated gene by performing the following searches:

- **Gene currently in G2P** [**https://www.ebi.ac.uk/gene2phenotype**](https://www.ebi.ac.uk/gene2phenotype)**?:** Check if the gene is already annotated in a G2P panel(s). If yes, annotate in which panel and what confidence category it has.
- **Gene currently in GenCC**[**https://search.thegencc.org/**](https://search.thegencc.org/)**?:** Check if the gene is currently present in the Gene Coalition Consortium (GenCC) database. If yes, record disease association and confidence category(s).

### 5. Clinical phenotype

The clinical phenotype is critical to determine the confidence level for a given gene-disease association during discussion at the curation meeting. For that purpose, the most relevant phenotypes observed in the disease are summarised as free text in the Clinical Phenotype section.

Then, for each publication, record the reported clinical phenotypes, including the following information when available:

- Proportion of individuals reported with a given phenotypic feature (i.e. variable expressivity)
- Evidence of incomplete penetrance
- If a phenotype is clinically distinctive and/or consistent

Evidence of associations between a particular phenotype, and/or severity/progression with specific variants or protein regions/domains

This information will be recorded as HPO terms in the G2P database.

### 6. Genotype

#### 6.1. Allelic requirement

This is a mandatory and unique field.

The allelic requirement reported in the publication for the gene-disease association is captured using the Human Phenotype Ontology (HPO) allelic requirement terms in the **Allelic requirement** table.

Allelic requirement is unique for each G2P record (gene-disease association). If the publication provides evidence supporting more than one allelic requirement for the gene-disease association, the curator will create a new record to annotate each gene-disease association allelic requirement independently (e.g. monoallelic_autosomal for monoallelic variants and biallelic_autosomal for biallelic variants).

In the case of monoallelic and biallelic variants, this rule applies to both scenarios, when the phenotypes associated with the monoallelic and biallelic variants are considered a similar disease entity or a distinct disease entity. For example, FBN1-Marfan syndrome caused by monoallelic variants and FBN1-Marfan syndrome caused by biallelic variants are annotated in two independent curation templates, as two independent records.

In the case of X-linked conditions, those usually penetrant in males and recessive in females may be recorded as monoallelic_X_hemizygous. X-linked diseases where heterozygous females and hemizygous males have similar phenotypes, for example in relation to SHOX and SMC1A variants, are recorded as monoallelic_X_heterozygous. However, it is recognized that this distinction may be difficult in practice, and separate entries can be used after discussion by the curation group.

For details, see Appendix Table 1. HPO allelic requirement terms.

#### 6.2. Cross cutting modifier

Use the HPO Inheritance qualifier terms in the **Cross-cutting modifier** table to annotate additional information reported in the publication that is relevant to the gene-disease inheritance.

**Potential secondary finding** is primarily used when there is evidence of a variable age of onset. For example, if a disorder is appropriate for DDG2P with onset in infancy, but older age of onset is also reported.

Use the **Restricted mutation set** checkbox when the variants associated with the curated disease are restricted, for example, to a protein domain/region or gene exon, or to single recurrent variants (usually <=3). This flag should not be used where recurrent variant(s) are included in a larger variant set with no identifiable pattern.For details, see Appendix Table 2. HPO inheritance qualifier terms.

6.3. Types of variants reported

This is a mandatory field.

Record all the types of variants associated with the curated gene-disease pair reported in the publication in the **Types of variants reported** table**.**

Use the second column to record if frameshift/nonsense and splice variants are predicted or proven to undergo or escape nonsense-mediated mRNA decay (NMD) (**Comment on NMD triggering/escaping**). It may be helpful here to look at the DECIPHER protein view “Predicted NMD Escape” track here. This column may also be used to record if variants are located in specific domains or regions and that location is relevant to interpret the variant effect and mechanism. For example, for missense variants clustering in a particular domain.

In addition, if the publication reports it, use the De novo and inherited columns to record variance inheritance. If no information is available, use the Unknown inheritance option.

For details, see Appendix Table 3. Sequence Ontology (SO) types of variant terms.

#### 6.4. DECIPHER Protein View

The DECIPHER Protein view ([www.deciphergenomics.org](http://www.deciphergenomics.org)) is used to allow curators to interpret the individual-level gene-disease information from the curated publications, into a wider molecular and genetic context, including:

- Molecular/gene-level data: exon boundaries, predicted NMD escape regions, conservation levels, missense constraint and protein domains;
- Disease-associated/diagnostic variation from DECIPHER and ClinVar (including reported pathogenicity)
- Population-level normal variation from gnomAD.

Include a snapshot of the DECIPHER protein view version used into the **DECIPHER Protein View** section for reference during the discussion at the curation meeting and future record updates. It is preferable to use the live DECIPHER website for curation, to utilise the interactive interface.

Information from the protein view needs to be interpreted in the context of the reported gene-disease association. For example, if missense variants are reported, is there missense constraint in the relevant region of the gene? What is the background variation (missense or LoF depending on reported variants) from gnomAD? If biallelic variants are reported, are there similar (missense or LoF as relevant) homozygous variants in gnomAD? If LoF variants are reported with no expression data, are these in the Predicted NMD Escape track?

#### 6.5. Variant consequence per allele for relevant allelic requirement

This is a mandatory field.

Use this section to record the consequence of the reported variants at the protein (for protein-coding genes) or the RNA (for non-protein coding genes), per allele.

For a given allelic requirement, Sequence Ontology (SO) terms for the functional effect of the variant are used.

Information based on computational or biological prediction is recorded as Inferred. Information based on experimental biological functional studies provided by the article is recorded as Evidence.

Examples:

- Inferred: when a variant is predicted to result in nonsense mediated decay; computational model of protein structure.
- Evidence: experiment showing absent protein expression by Western Blot or immunostaining.

For details, see Table 4. Sequence Ontology (SO) functional effect variant terms.

### 7. Mechanism

This is a mandatory and unique field.

G2P aims to capture the specific molecular mechanisms underlying gene-disease associations. To record the mechanism of the disease, first use one of the 5 broad categories in the Mechanism table. These include ‘Loss of function’, ‘Dominant negative’, ‘Gain of function’, ‘Undetermined non-loss-of-function’ and ‘Undetermined’. Undetermined category is used when the mechanism is still unknown, or the evidence supporting the proposed mechanism is not strong enough. The Mechanism table in the curation template includes a detailed description of the available categories.

Information based on computational or biological prediction is recorded as Inferred (eg. algorithms predicting the molecular mechanism of a variant/protein, modelling 3D protein structure) . Information based on experimental biological functional studies provided by the article is recorded as Evidence (eg. transgenic/knock in model organisms, biochemical function assays).

**Example:**

- **SLC37A4-related congenital disorder of glycosylation with liver dysfunction**
- **Mechanism:** Other GOF - inferred
- The c.1267C>T (p.Arg423∗) in SLC37A4 is predicted to delete the last seven amino acids from the cytoplasmic tail. PMID 33964207 shows mutated transporter is mislocalised – not in Golgi. Dose dependent morphological and functional change in Golgi. 32884905 also demonstrates mislocalisation. No definitive evidence of effect on wild type protein therefore unlikely dominant negative. Note biallelic null variants cause disease so unlikely loss of function.

#### 7.1. Synopsis of mechanism

When the publication provides additional information about the underlying molecular mechanisms of a ‘Loss of function’, ‘Dominant negative’ or ‘Gain of function’ disease mechanism, use the terms in the Synopsis of mechanism table to capture that information.

In addition to the articles being curated, check MaveDB, a public repository for datasets from Multiplexed Assays of Variant Effect (MAVEs), for assays relevant for the specific gene. If available, these need to be assessed as to whether the assay method is relevant to the reported disease mechanism.

When reviewing the experimental/functional information provided in the article about the disease mechanism, it is critical to capture the genetic and biological models used for the functional assays. This information is needed to assess the relevance of the functional data for the specific gene-disease association and will be used during the curation meeting discussions.

Examples:

- Studies of the whole gene versus disease specific variants function
- Cell/tissue involved in the disease pathogenesis
- Biological models replicating the disease molecular mechanisms and/or phenotypes

Information based on computational prediction is recorded as Inferred. Information based on experimental biological functional studies provided by the article is recorded as Evidence.

Additional details supporting the variant consequences and mechanism classification that are relevant for the assessment of the gene-disease association can be recorded as free text in the two available text boxes.

### 8. Additional comments

This section is used to capture additional information about:

- The gene mutational landscape: what other variants (particularly, Pathogenic and Likely Pathogenic) have been reported (sources DECIPHER, ClinVar). These can be found in the DECIPHER protein view.
- Associations of the gene to disease in other resources, for example Genomics England PanelApp
- Gene population level normal variation (source gnomAD, other relevant reference population genomics databases). This can be found in the DECIPHER protein view.

### 9. Disease entity

#### 9.1. Disease name

This is a mandatory and unique field.

Disease names are discussed and agreed on by the curation team. G2P follows the dyadic naming system as "GENE SYMBOL-related phenotype descriptor" (for details see [Biesecker et al. 2021](https://pubmed.ncbi.nlm.nih.gov/33417889/)).

##### 9.1.1. Existing disorders

For disorders already named in internationally recognised curation resources (reference resources are OMIM and MONDO), use the section Discussion of current gene-disease name and additional information, if applicable Synonyms to capture the current gene-disease names in use. If an existing disease name is compatible with the dyadic approach, use that name and adapt if necessary by adding ‘Gene symbol-related’ to match G2P preferred format.

Preferentially, a precise clinically relevant phenotypic name is used, for example **AMOTL1-related orofacial clefting, cardiac anomalies, and tall stature**. However, in other cases the disease can follow well established eponymous names, for example **PTPN11-related Noonan syndrome**. Where available, cross reference to OMIM Morbid ID and/or MONDO ID to facilitate integration of G2P data with external collaborators and resources.

##### 9.1.2. New disorders

For new disorders not yet named in internationally recognised curation resources, propose a disease name by using the HGNC gene symbol together with the most relevant clinical phenotypes observed. For example, **RNU4-2 related neurodevelopmental disorder with microcephaly and seizures**. When creating a G2P record for a new gene-disease relationship not yet found in OMIM and/or MONDO, flag the G2P record to request the creation of that disease term in MONDO.

#### 9.2. Panel

In the Panel table, record which G2P panels are relevant for the reported phenotype (DD, Cancer, Eye, Neonatal, Obesity, Paed Neuro, Skeletal, Skin). Assignment to panels should be agreed at the curation meeting. The clinical curation leads of the other G2P panels can be contacted to confirm the inclusion of the gene-disease association in the respective panel if necessary.

#### 9.3. Agreed confidence category

Assign a confidence category to the gene-disease association based on the curated information in the Agreed confidence category table. This confidence score indicates the likelihood that the gene-disease association to be true.

For details, see Table 5. Description of G2P confidence categories.

If the curation done was to review a gene-disease association already existing in G2P and the curation results in an upgrade or downgrade of the existing confidence score, use the **Changed from** table to record the original Confidence category.

### Appendix

#### Table 1. HPO allelic requirement terms

**Allelic requirement/Mendelian inheritance terms (HP:0034345)**

| **allelic requirement term** | **Mendelian inheritance** | **HPO id** | **G2P definition** |
| --- | --- | --- | --- |
| monoallelic_autosomal | Autosomal Dominant | [HP:0000006](https://hpo.jax.org/browse/term/HP:0000006) | Plausible disease-causing mutations on an autosomal chromosome identified on one allele in all or the vast majority of with specific disorder. |
| biallelic_autosomal | Autosomal Recessive | [HP:0000007](https://hpo.jax.org/browse/term/HP:0000007) | Plausible disease-causing homozygous or compound heterozygous mutations identified on both alleles in the autosomal chromosome. |
| monoallelic_X_heterozygous | X-linked dominant | [HP:0001423](https://hpo.jax.org/browse/term/HP:0001423) | Plausible disease-causing mutations identified in one copy of the X chromosome in females as a cause of a specific disease, include disorders where heterozygous females and hemizygous males are similarly affected e.g SMC1A mutations. |
| monoallelic_X_hemizygous | X-linked recessive | [HP:0001419](https://hpo.jax.org/browse/term/HP:0001419) | Plausible disease-causing mutations identified on the X chromosome in a male as a cause of a specific disease, the disorder being predominantly recessive in female carriers. |
| monoallelic_Y_hemizygous | Y-linked | [HP:0001450](https://hpo.jax.org/browse/term/HP:0001450) | Plausible disease-causing mutations identified in an allele found in the Y chromosome. The Y chromosome is passed from father to son as this mutation may affect only males. |
| mitochondrial | Mitochondrial | [HP:0001427](https://hpo.jax.org/browse/term/HP:0001427) | Plausible disease-causing mutations identified on mitochondrial DNA where homoplasmy or heteroplasmy are associated with a specific disorder. |
| monoallelic_PAR | PAR dominant | [HP:0034340](https://hpo.jax.org/browse/term/HP:0034340) | Plausible disease-causing mutations identified in an allele found in the pseudoautosomal regions. Inheritance is not strictly sex-linked. (PAR=pseudoautosomal region). |
| biallelic_PAR | PAR recessive | [HP:0034341](https://hpo.jax.org/browse/term/HP:0034341) | Plausible disease-causing homozygous or compound heterozygous mutations identified on both alleles found in the pseudoautosomal regions. Inheritance is not strictly sex-linked. |

#### Table 2. HPO inheritance qualifier terms.

**Cross-cutting modifier/Inheritance qualifier (HP:0034335)**

| **Inheritance Modifier** | **HPO id** | **G2P definition** |
| --- | --- | --- |
| Typically de novo | [HP:0025352](https://hpo.jax.org/browse/term/HP:0025352) | Plausible disease causing mutations that occur post zygotically (formation of gametes). |
| Typically mosaic | [HP:0001442](https://hpo.jax.org/browse/term/HP:0001442) | Plausible disease causing mutations identified on one allele in a proportion of cells with the others being wild-type. |
| Typified by incomplete penetrance | [HP:0003829](https://hpo.jax.org/browse/term/HP:0003829) | Plausible disease-causing mutations from an apparently unaffected parent on several occasions.. |
| Imprinted region | [HP:0034338](https://hpo.jax.org/browse/term/HP:0034338) | Plausible disease-causing mutations identified in one allele in which the allele's parental origin determines the disease/phenotype exhibited. |
| Displays anticipation | [HP:0003743](https://hpo.jax.org/browse/term/HP:0003743) | A type of autosomal dominant inheritance involving a gene that exhibits anticipation, the increase in severity and/or an earlier age of onset in subsequent generations. |
| **Additional terms** |  |  |
| Potential secondary finding (including ACMG Secondary Findings and/or late onset conditions) | - | Plausible disease-causing mutations within, affecting or encompassing the coding region of a single gene identified in multiple (>3) unrelated cases/families with both the relevant disease (RD) and an incidental disorder. |
| Restricted mutation set | **-** | Restricted repertoire of mutations. For example, diseases which are associated with a single recurrent variant, or variants only found in a particular protein domain. |

####

#### Table 3. Sequence Ontology (SO) type of variant terms.

| **TYPE OF VARIANT** | **SO term ID** | **Definition** |
| --- | --- | --- |
| **Frameshift & nonsense variants** |  |  |
| frameshift_variant | [SO:0001589](http://sequenceontology.org/browser/current_release/term/SO:0001589) | A sequence variant which causes a disruption of the translational reading frame, because the number of nucleotides inserted or deleted is not a multiple of three. |
| stop_gained | [SO:0001587](http://sequenceontology.org/browser/current_release/term/SO:0001587) | A sequence variant whereby at least one base of a codon is changed, resulting in a premature stop codon, leading to a shortened polypeptide. |
| **Splice variants** |  |  |
| splice_region_variant | [SO:0001630](http://sequenceontology.org/browser/current_release/term/SO:0001630) | A sequence variant in which a change has occurred within the region of the splice site, either within 1-3 bases of the exon or 3-8 bases of the intron. |
| splice_acceptor_variant | [SO:0001574](http://sequenceontology.org/browser/current_release/term/SO:0001574) | A splice variant that changes the 2 base region at the 3' end of an intron. |
| splice_donor_variant | [SO:0001575](http://sequenceontology.org/browser/current_release/term/SO:0001575) | A splice variant that changes the 2 base pair region at the 5' end of an intron. |
| **Missense & inframe variants** |  |  |
| missense_variant | [SO:0001583](http://sequenceontology.org/browser/current_release/term/SO:0001583) | A sequence variant, that changes one or more bases, resulting in a different amino acid sequence but where the length is preserved. |
| inframe_insertion | [SO:0001821](http://sequenceontology.org/browser/current_release/term/SO:0001821) | An inframe non synonymous variant that inserts bases into in the coding sequence. |
| inframe_deletion | [SO:0001822](http://sequenceontology.org/browser/current_release/term/SO:0001822) | An inframe non synonymous variant that deletes bases from the coding sequence. |
| **Other variants** |  |  |
| intergenic_variant | [SO:0001628](http://sequenceontology.org/browser/current_release/term/SO:0001628) | A sequence variant located in the intergenic region, between genes. |
| intron_variant | [SO:0001627](http://sequenceontology.org/browser/current_release/term/SO:0001627) | A transcript variant occurring within an intron. |
| synonymous_variant | [SO:0001819](http://sequenceontology.org/browser/current_release/term/SO:0001819) | A sequence variant where there is no resulting change to the encoded amino acid. |
| Stop lost | [SO:0001578](http://sequenceontology.org/browser/current_release/term/SO:0001578) | A sequence variant where at least one base of the terminator codon (stop) is changed, resulting in an elongated transcript. |
| Whole/partial gene deletion | - | An deletion which includes contiguous nucleotides of a gene. It can include a part or the whole gene. |
| Whole/partial gene duplication | - | An insertion which derives from, or is identical in sequence to, nucleotides present in a gene. It can include a part or the whole gene. |
| short_tandem_repeat_change | [SO:0002161](http://sequenceontology.org/browser/current_release/term/SO:0002161) | A sequence variant where the copies of a short tandem repeat (STR) feature are either contracted or expanded. |
| start_lost | [SO:0002012](http://sequenceontology.org/browser/current_release/term/SO:0002012) | A codon variant that changes at least one base of the canonical start codon. |
| ncRNA | [SO:0000655](http://sequenceontology.org/browser/current_release/term/SO:0000655) | An RNA transcript that does not encode for a protein rather the RNA molecule is the gene product. |
| **Variants in regulatory regions** |  |  |
| 5_prime_UTR_variant | [SO:0001623](http://sequenceontology.org/browser/current_release/term/SO:0001623) | A UTR variant of the 5' UTR. |
| 3_prime_UTR_variant | [SO:0001624](http://sequenceontology.org/browser/current_release/term/SO:0001624) | A UTR variant of the 3' UTR. |
| regulatory_region_variant | [SO:0001566](http://sequenceontology.org/browser/current_release/term/SO:0001566) | A sequence variant located within a regulatory region. |

#### Table 4. Sequence Ontology (SO) functional effect variant terms.

**Functional effect variant (SO:0001536)**

| **Term** | **SO term ID** | **Definition** |
| --- | --- | --- |
| Altered_gene_product_level | [SO:0002314](http://sequenceontology.org/browser/current_svn/term/SO:0002314) | A sequence variant that alters the level or amount of gene product produced. This high level term can be applied where the direction of level change (increased vs decreased gene product level) is unknown or not confirmed. |
| - Decreased_gene_product_level | [SO:0002316](http://sequenceontology.org/browser/current_svn/term/SO:0002316) | Decreased gene product level, decreased transcription level, decreased_transcription_level, reduced gene product level, reduced transcription level, reduced_gene_product_level, reduced_transcription_level |
| - Absent_gene_product | [SO:0002317](http://sequenceontology.org/browser/current_svn/term/SO:0002317) | A sequence variant that results in no gene product. |
| - Increased_gene_product_level | [SO:0002315](http://sequenceontology.org/browser/current_svn/term/SO:0002315) | A variant that increases the level or amount of gene product produced. |
| Altered_gene_product_sequence | [SO:0002318](http://sequenceontology.org/browser/current_svn/term/SO:0002318) | A sequence variant that alters the sequence of a gene product. |
| Uncertain | - | A sequence variant for which the functional consequence it is not known or uncertain |

#### Table 5. Description of G2P confidence categories.

| **Term** | **Definition** |
| --- | --- |
| Definitive | The role of this gene in this particular disease has been repeatedly demonstrated in both the research and clinical diagnostic settings, and has been upheld over time (at least 2 independent publications over 3 years' time). No convincing evidence has emerged that contradicts the role of the gene in the specified disease.  Used for clinical reporting. |
| Strong | The role of this gene as a monogenic cause of disease has been repeatedly and independently demonstrated providing very strong convincing evidence in humans and no conflicting evidence for this gene's role in this disease.  Used for clinical reporting. |
| Moderate | There is moderate evidence in humans to support a causal role for this gene in this disease with no contradictory evidence. The body of evidence is not large (e.g possibly only one key paper) but appears convincing enough that the gene-disease pair is likely to be validated with additional evidence in the near future.  Used for clinical reporting. |
| Limited | Little human evidence exists to support a causal role for this gene in this disease, but not all evidence has been refuted. For example, there may be a collection of rare missense variants in humans but without convincing functional impact, segregation data that could either arise by chance (e.g across one or two meioses) or does not implicate a single gene, or functional data without direct recapitulation of the phenotype. Overall, the body of evidence does not meet contemporary criteria for claiming a valid association with disease. The majority are probably false associations.  Not used for clinical reporting. |
| Disputed | Although evidence has been reported, other evidence of equal weight disputes the claim. |
| Refuted | There has been an assertion of a gene-disease association in the literature, but new valid evidence has arisen that refutes the entire original body of evidence. |
